# Characterization and modeling of diffusion kinetics of rosemary oleoresin extract from gellan gum-based film

**DOI:** 10.1007/s13197-023-05826-9

**Published:** 2023-09-13

**Authors:** Ru Wei Teoh, Adeline Su Yien Ting, Yin Yin Thoo

**Affiliations:** School of Science, Monash University Malaysia, Bandar Sunway, 47500 Subang Jaya, Selangor Malaysia

**Keywords:** Active film, Antioxidant, Barrier properties, Rosemary, Zein

## Abstract

Fresh food products are highly prone to oxidation and microbial attack, rendering them unsuitable for consumption. Thus, active food packaging was developed to protect and prolong food shelf-life. Zein/gellan gum (GG) based active film is developed by incorporating rosemary oleoresin extract (ROE) (0–20%). The films were characterized by their barrier and antioxidant properties. The release behavior of ROE in fatty and hydrophilic food stimulants was investigated via mathematical modeling. The active films incorporated with 20% ROE have significantly higher oxygen barrier and oxygen transfer is reduced by 20% compared to the control. A tortuous path is created with ROE, which impedes oxygen movement across the film. ROE addition improved water resistance performance by reducing the active film swelling ratio by 31%. This improvement is attributed to the hydrophobic nature of ROE. FTIR shows that the interaction between ROE and the active film is primarily hydrogen bonding and electrostatic interactions. Active film exhibits excellent antioxidant activity, with high TPC, DPPH scavenging activity, and FRAP. Mathematical modeling revealed a higher diffusivity (*D*) of ROE in fatty food stimulants at 24 °C, attributed to high polarity and solubility in fatty food stimulants. Overall, this active film has an excellent antioxidant effect and could potentially be used as food packaging for high-fat food products to prevent oxidation.

**Graphical abstract:**

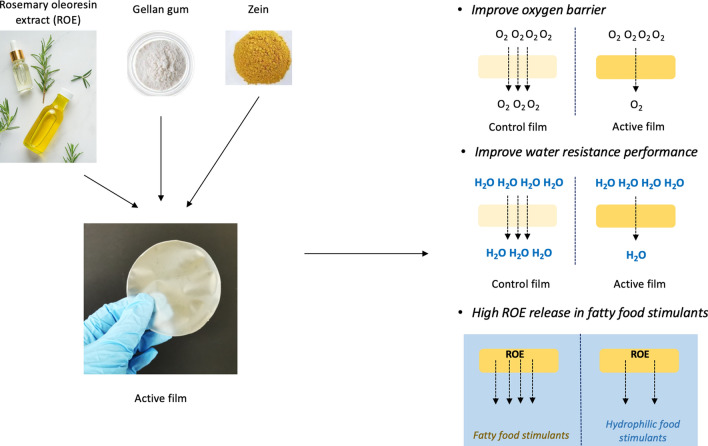

## Introduction

Food products are prone to deterioration over time, such as oxidation and microbial contamination. These will affect food products' quality and safety, rendering them unsuitable for consumption. Therefore, developing active food packaging that exhibits antioxidant and antimicrobial properties is essential in prolonging food shelf-life. Besides, the constituents of active food packaging should be sourced from renewable and biodegradable materials, such as natural biopolymers or extracts. This will minimize environmental pollution and reduce the dependency on plastic-based materials.

Among many natural biopolymers, gellan gum (GG) received much interest recently. GG has good gelling and film-forming properties, rendering it a highly promising material for applications in the food, and medical industries (Saha and Bhattacharya 2010). GG is a water-soluble, anionic polysaccharide produced by *Sphingomonas elodea*. GG has many acyl groups and is known as high-acyl GG (HAGG) in its native form. GG is frequently utilized in food applications as a thickener and gelling agent to improve food product quality. Despite having these benefits, the weak water resistance and gas barrier properties limits the application in food packaging film. Thus, GG is blended with other inorganic nanoparticles or naturally-derived biopolymers to enhance barrier and water-resistance properties, such as chitosan, pectin and cellulose nanocrystal. The overall water resistance of GG/agar composite film is improved after adding montmorillonite clay (Lee et al. 2019). Adding alginate into the film also results in a synergistic effect, which enhances the water resistance of composite film on paper cups for hot drinks (Zhang et al. 2017).

Zein is a hydrophobic plant storage protein isolated from the endosperm of corn kernels. The hydrophobic nature of zein, which makes it insoluble in water but soluble in alcohol, can be attributed to the significant content of hydrophobic amino acids. This contributes to excellent water-resistance performance and low gas permeability when blended with other biopolymers in food packaging (Ahammed et al. 2020). Incorporation of zein in chitosan film minimise the water vapor transmission across film, reduced the water absorbency and moisture content of film (Brodnjak and Tihole 2020). In addition, blending zein with pre-gelatinized maize starch films improves mechanical and water resistance properties (Teklehaimanot et al. 2020). These findings suggest the potential of zein in improving water resistance and barrier properties of polysaccharide film.

Rosemary oleoresin extract (ROE) can be obtained naturally through the leaves and stems of the rosemary plant. ROE contains a wide variety of polyphenol compounds, and the main compounds include carnosic acid, carnosol, rosmarinic acid, rosmaridiphenol, rosmanol, and hesperidin. These compounds contribute to the potent antioxidant activity and allow ROE to be used in various food applications, such as preventing edible oil, meat products, and beverages from oxidation. The incorporation of rosemary extract enhanced oxidative stability of cottonseed, soybean and rice bran oil, particularly reducing primary oxidation throughout the storage period (Yang et al. 2016). Recently, the ROE was also applied to food packaging. The polylactic acid-based food packaging film embedded with rosemary extract presents a high antioxidant activity and effectively scavenges free radicals (Vasile et al. 2019).

Release kinetics is crucial to active food packaging, as the active compound in active packaging can potentially migrate into food products and influence shelf-life. Thus, it was widely studied on different food stimulants, temperatures, film types, and salt concentrations to understand the release behavior (Benbettaïeb et al. 2020). However, there still needs to be a greater understanding of the diffusion coefficient of active compounds in active packaging. Several studies have discussed the antioxidant capabilities of GG-based active film, but there is limited study investigating the release behavior of ROE from GG-based active film into fatty and hydrophilic food stimulants. Furthermore, the impact of ROE on the water resistance and barrier properties of GG-based film has not been explained yet. Therefore, the antioxidant properties of zein/GG film incorporated with ROE is determined in this study. Furthermore, the diffusion coefficient (*D*) of ROE in fatty and hydrophilic food stimulants will be determined through mathematical modeling and performed in a room and at refrigeration temperatures. The effect of ROE on water resistance and a gas barrier was evaluated through swelling ratio, water contact angle, and oxygen permeability of active film. Nano-analytical tools, AFM, FESEM and ATR-FTIR spectroscopy were used to elucidate the underlying interaction between ROE and zein/GG film.

## Materials and methods

### Materials

HAGG (Kelcogel LT100, CP Kelco) and ROE were obtained as a gift from Brenntag Sdn. Bhd. (Shah Alam, Malaysia) and Hainan Super Biotech Co., Ltd (Hainan, China), respectively. Zein, anhydrous Calcium Chloride (CaCl_2_), sodium carbonate (Na_2_CO_3_), glycerol, Folin-Ciocalteau’s reagent, 2,3,5-Triphenyltetrazolium chloride (TPTZ), Iron(III) chloride (FeCl_3_), 2,2-diphenyl-1-picryl-hydrazine-hydrate (DPPH), trolox and gallic acid were purchased from Sigma-Aldrich (St. Louis, MO. USA).

### Preparation of active film

Solvent casting method was used to fabricate the active films. 100 mL distilled water was mixed with GG powder (0.5 g) at 80 °C, under continuous stirring for 20 min, to ensure GG powder was completely dissolved. After 20 min of mixing, 0.02 g (4% w/w of GG powder) of anhydrous CaCl_2_ was added into the GG solution and continued stirring for 1 h. Then, the mixture was added with 300 μL (0.3% v/v of GG solution) of glycerol and stirred for 20 min. Before adding the zein/ROE mixture into GG solution, 0.5 g of zein powder was added into 100 mL ethanol/water solvent (80:20, v/v) at room temperature and stirred continuously for 2 h to ensure complete dissolution of zein powder. Zein solution was then added into GG solution dropwise to ensure consistency at 80 °C, stirring at 60 × g for 30 min (Wang et al. 2017). The concentrations of zein and ROE were 5%, 10%, 15%, and 20% (dry weight of zein: dry weight of GG, w/w). The zein/ROE/GG mixtures were then carefully poured onto Petri dishes, with 50 mL for each petri dish, and oven dried at 45 °C. The active film was formed after 24 h, when the water in the zein/GG mixture was completely evaporated.

### Physical characteristics of active film

#### Swelling ratio

Active films were sampled into square pieces (2 cm × 2 cm), and the weight was recorded before immersing in distilled water for 24 h. Then, the active films were weighed again after 24 h to determine of swelling ratio. Five replicates were performed for each ROE concentration. The swelling ratio of active films was calculated through the following equation Eq. (1):

1 $${\text{Swelling}}\;{\text{ratio}} \left( \% \right) = \frac{{W_{{\text{f}}} - W_{{\text{i}}} }}{{W_{{\text{i}}} }} \times 100$$

In the above equation, *W*_f_ and *W*_i_ were the final and initial weights of active film, respectively.

#### Water contact angle (WCA) and oxygen permeability (OP)

Sessile drop method was used to measure the WCA on active film, through the ramé-hart Model 250 goniometer (ramé-hart, Succasunna, New Jersy, USA). Active films were placed on top of a glass slide, and a drop of distilled water (4 μL) was deposited in five random places on each composite film. The WCA and images were taken every 1 min, for 5 min and recorded using DROPimage Advanced (ramé-hart, Succasunna, New Jersy, USA) software.

Sunflower oil (15 mL) was added to the crystallizing dish, and the opening was sealed with composite film. The edge of the crystallizing dishes was then sealed completely using Vaseline and parafilm. Negative control with the crystallizing dish without active films was used in this study. All test sets were incubated at 50 °C for 7 days and expressed as peroxide values (P; meq hydroperoxide/kg oil), according to the AOCS official method (Cd 8-53).

### Flim morphology

#### Field-emission scanning electron microscope (FESEM)

FESEM (Hitachi SU8010, Hitachi, Minato-ku, Tokyo, Japan) was used to study the morphology of the active film. Active films incorporated with four varied ROE concentrations were sampled into small squares (3 mm × 3 mm) and attached to the specimen stage using double-stick carbon tape. Then, all films were platinum-sputter-coated. All composite films were examined with an accelerating voltage of 2.0 kV and × 5.00 k magnification.

#### Atomic force microscope (AFM)

AFM was used to study the 3D surface topography of active films. AFM (Multimode 8, Bruker, Billerica, Massachusetts, USA) with tapping mode and silicon nitride probes of resonance frequency at 70 kHz and a spring constant of 0.4 N/m was used in this study. The composite film samples of small squares (3 mm × 3 mm) was used. Two areas (5 μm × 5 μm) of small squares were scanned at a speed of 0.8Hz with a resolution of 512 × 512 pixels.

### Molecular interaction of active film

ATR-FTIR spectroscopy analysis was done to understand the molecular interaction in active film. The spectra of the active film were recorded on Spectrum Two FT-IR spectrometer (PerkinElmer, Waltham, Massachusetts, USA). All active films were run using an ATR accessory, in the range of 4000–400 1/cm with 32 scans and a resolution of 4 1/cm.

### Antioxidant activity

#### Total phenolic content (TPC)

The TPC of active film incorporated with ROE was determined through Folin-Ciocalteu (FC) method. Active film samples (0.2 g) were immersed in 95% ethanol to obtain the film extract, at room temperature for 24 h. Film extracts (0.75 mL) were mixed with distilled water (3.25 mL), followed by the addition of 10% v/v FC reagent (0.4 mL) and incubated for 5 min. After that, 7.0 g/L of sodium carbonate solution (1 mL) was added to the mixture and incubated for 30 min, in the dark (Lee et al. 2013). UV-vis spectrophotometer was used to record the absorbance (765 nm). A standard curve of gallic acid (GA) was obtained with a concentration from 0 to 0.12 mg/ml and *R*^2^ = 0.9968. The results were expressed as mg GA equivalent/g of the film (mg GAE/g of the film).

#### DPPH-radical scavenging activity

DPPH-radical scavenging activity of active film was determined spectrophotometrically. Film extract (0.2 mL) was mixed with 40 μM of ethanolic DPPH solution (3.8 mL) and vortex for 30 s. After incubating the mixture for 30 min, in the dark, UV-vis spectrophotometer was used to record the absorbance (517 nm). A standard curve of Trolox was obtained with a concentration from 0 to 0.7 mM and *R*^2^ = 0.9912. The results were expressed as mM Trolox equivalent/g of the film (mM TE/g of the film).

#### Ferric reducing antioxidant power (FRAP) assay

FRAP assay was performed spectrophotometrically to determine the antioxidant power of the active film. Before analyzing film extracts, FRAP reagent solution was prepared with 20 mM FeCl_3_ solution, 10 mM TPTZ in 40 mM HCL solution and 300 mM acetic buffer, in a 1:1:10 ratio, respectively (Lee et al. 2013). The prepared FRAP reagent solution (3.6 mL) was then added into film extract (0.18 mL) and incubated for 8 min, in the dark. After that, UV-vis spectrophotometer was used to record the absorbance (593 nm). A standard curve of gallic acid (GA) was obtained with a concentration from 0 to 0.05 mg/ml and *R*^2^ = 0.9848. The results were expressed as mg GA equivalent/g of the film (mg GAE/g of the film).

### Release test of active film in food stimulants

The release study was performed by immersing active film (0.2 g) into two food stimulants, hydrophilic (10% ethanol) and fatty (95% ethanol) food stimulants. Film extracts (0.75 mL) were obtained every 3 h, for the first 24 h, and every 24 h for the subsequent 3 days (Cui et al. 2021). The film extracts were used to determine the amount of ROE released at a specific time point through performing TPC (same procedure as *above*). The results were expressed as mg GAE/kg food stimulants. This process was repeated at 24 °C (room temperature) and 4 °C (refrigeration temperature).

### Determination of diffusion coefficient (*D*) through mathematical modelling

*D* of ROE in active film in different food stimulants was calculated using the analytical solution of Fick’s second law (Eq. (2)):

2 $$\frac{{\partial C_{{\text{F}}} }}{\partial t} = D\frac{{\partial^{2} C_{{\text{F}}} }}{{\partial x^{2} }}$$

where *C*_F_ represents the concentration of an antioxidant compound in active film over time, *t*, $x$ represents the film thickness, and *D* represents the diffusion coefficient of antioxidants in active film. This model (Eq. (3)) determines the diffusivity of a solute from a limited volume of the plane sheet into a limited volume of solution. Thus, this model is appropriate for this study (Benbettaïeb et al. 2020). Whereby, ROE diffused from the active film (plane sheet) into a fixed volume of food stimulants. Moreover, a few assumptions were made for this model (Eq. (3)):

1. No boundary layers affect ROE transfer (ROE travels out from the active film at the same rate as it travels into food stimulants). The liquid side of active films is not affected by mass transfer resistance.
2. The diffusion of ROE is in a single direction. Where ROE only diffuses from the center to the surface of the active film.
3. Diffusivity is independent of concentration and time.

3 $$\frac{{C_{{\text{S,t}}} }}{{C_{{{\text{S}},\infty }} }} = 1 - \mathop \sum \limits_{n = 1}^{\infty } \frac{{2\alpha \left( {1 + \alpha } \right)}}{{1 + \alpha + \alpha^{2} q_{n}^{2} }}{\text{exp}}\left( { - \frac{{Dq_{n}^{2} t}}{{L^{2} }}} \right)$$

where *C*_S,t_ represents the concentration of antioxidants in food stimulants over time *t*, *C*_S*,∞*_ represents the concentration of antioxidants at equilibrium, and *L* represents film thickness. The *α* is determined via Eq. (4) that describes the ratio of the volume of food stimulants to the volume of active film. The *α* value is affected by a partition coefficient (*K*_FS_*)* that corresponds to the final fractional uptake of ROE (Eq. (5)).

4 $$\alpha = \frac{{V_{{\text{S}}} }}{{\left( {K_{{{\text{FS}}}} \times V_{{\text{F}}} } \right)}}$$

where *V*_S_ represents the volume of food stimulants, *V*_F_ represents the volume of active film. *K*_FS_ represents the partition coefficient of antioxidants between the film and food stimulants.

5 $$K_{{{\text{FS}}}} = \frac{{C_{{{\text{F}},\infty }} }}{{C_{{{\text{S}},\infty }} }}$$

where *C*_F*,∞*_ represents the antioxidant concentration in film at equilibrium, *C*_S*,∞*_ represents the food stimulants at equilibrium.

From Eq. (3), *q*_n_ was obtained from non-positive roots of Eq. (6):

6 $$\tan \left( {q_{{\text{n}}} } \right) = - aq_{{\text{n}}}$$

Equation (6) affects the reliability of the results. So, a more significant number of roots, 12 roots, were calculated for 0.01 ≤ *α* ≤ 1000 to achieve a more reliable result.

When the migration of antioxidants is low in a film to food stimulants, more roots were required to increase the accuracy, which makes Eq. (3) unsuitable in low migration conditions, particularly with α < 0.01. Therefore, Eq. (7) was used for hydrophilic food stimulants.

7 $$\frac{{C_{{\text{S,t}}} }}{{C_{{{\text{S}},\infty }} }} = \left( {1 + \alpha } \right)\left[ {1 - e^{\omega } {\text{erfc}}\left( {\omega^{0.5} } \right)} \right]$$

8 $$\omega = \frac{{D_{p} t}}{{\alpha^{2} L_{p}^{2} }}$$

where *C*_S,t_ represents the concentration of antioxidants in food stimulants at time *t*, *C*_S*,∞*_ represents the concentration of antioxidants in food stimulants at equilibrium, *D* diffusion coefficient, *L* represents half thickness of film.

Both of these models (Eqs. (3) and (7) were applied to the release test of active film in hydrophilic and fatty food stimulants to determine the diffusion coefficient of antioxidants in active film. The coefficient of determination (*R*^2^) was calculated to determine the fitting between experimental data and theoretical values. All modellings were performed using Matlab Software (The Mathworks, Natick, MA).

### Statistical analysis

Analysis done on active film in determining antioxidant activity, release test and physical characteristics were statistically analysed and compared using SPSS (SPSS Inc, Chicago, IL, USA) by one-way analysis of variance (ANOVA). Tukey’s test was performed to compare all the means, and significant differences were determined at *p* < 0.05.

## Results and discussions

3.1 Film morphology

**Fig. 1:**
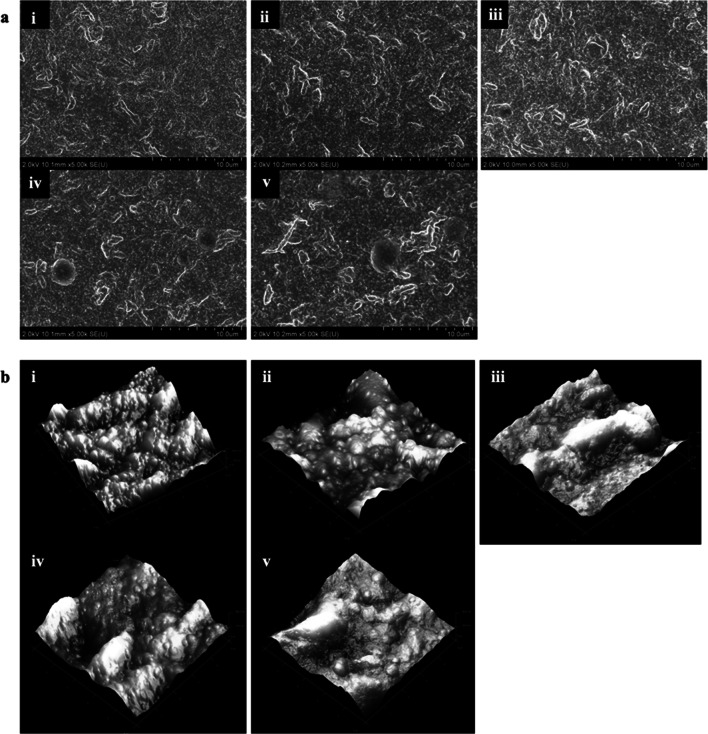
Morphology of active films incorporated with various ROE concentrations from 0% to 20% (i–v) evaluated through **a** FESEM at × 5.00 k magnification and **b** AFM with tapping mode

The morphology and 3D surface topography of active films were observed through FESEM and AFM, respectively. From Figure 1, the film surface without ROE addition appears to have aggregated GG granules. Besides that, smaller, round-shaped zein particles can also be seen and well-dispersed across the film surface. Similar observations were also found with AFM. This appearance remained consistent with no obvious apparent changes with 5 and 10% ROE addition. However, observable changes were detected for 15% ROE addition with the presence of ROE globule, which is believed to be ROE that has not interacted with zein. Moreover, the round-shaped zein particles start to become less distinct. With 20% ROE addition, the ROE globule becomes larger and some of the zein particles begin to aggregate and become larger round clumps. This is associated with the increase in ROE concentration and despite ROE binding with individual zein particles, they are more likely to aggregate in high-concentration environments (Liang et al. 2022). Images from AFM also showed that flat, round globules are present, believed to be free ROE that binds together and forms globules. Similar observations were also obtained when plant essential oil was loaded into chitosan nanoparticles and the particles were aggregated (Zhang et al. 2020).

### Molecular interaction of active film

**Fig. 2:**
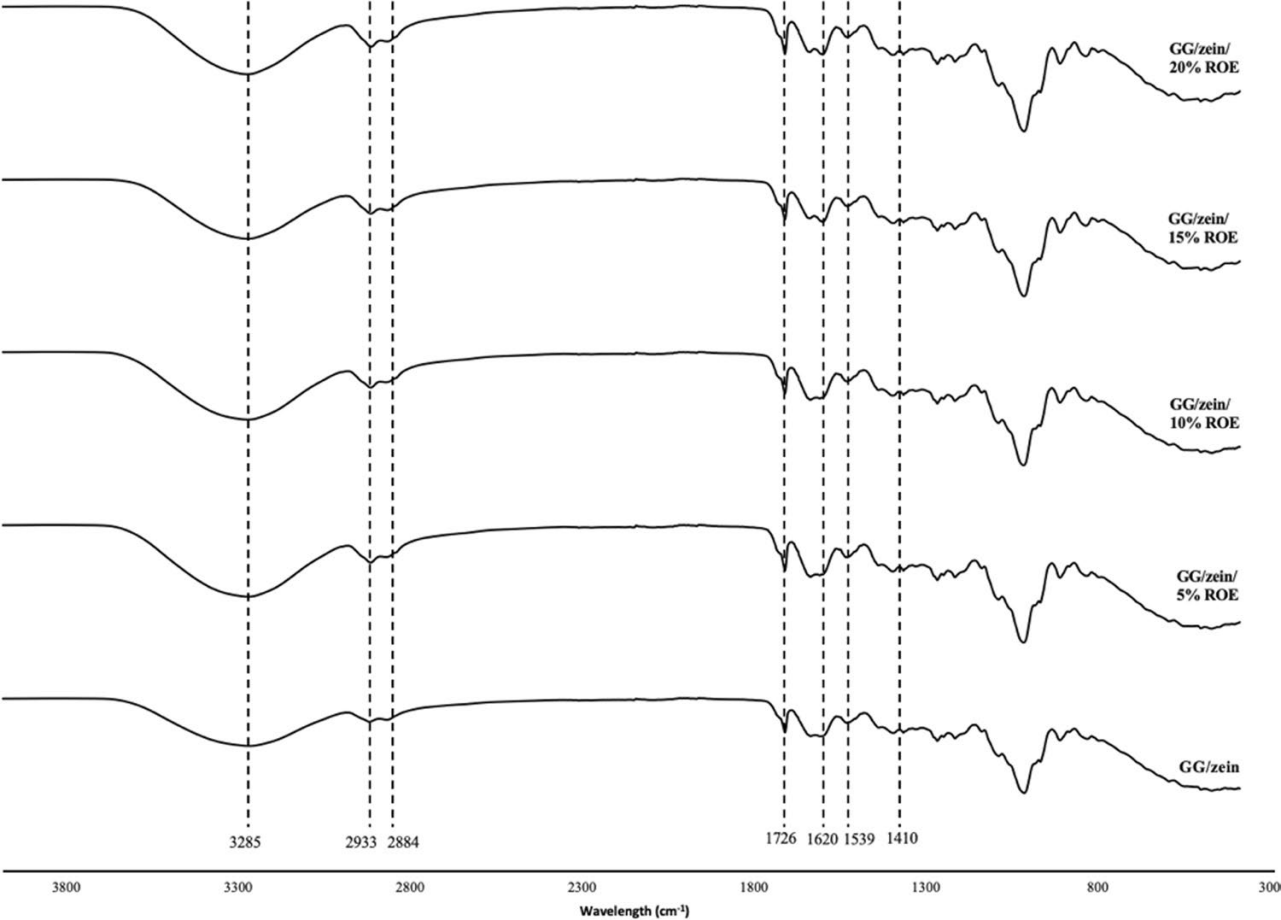
FTIR spectrum of active films incorporated with various ROE concentrations (0–20%) at 400–4000 1/cm

FTIR elucidated the interactions of functional groups within the active film, providing valuable information on intermolecular bonds. For a film without ROE addition (0% ROE), the peak at 3285 1/cm represents the O-H stretch from the hydroxyl group, specifically the hydrogen bond formed between GG and zein (Wang et al. 2017). As seen in Figure 2, the peaks at 2933 1/cm and 2884 1/cm represent the absorptions of the C–H stretch. Besides that, the peak at 1620 1/cm represents absorptions of C=O stretch from carboxylate. While both peaks, 1620 cm^-1^ and 1539 1/cm, are electrostatic interactions between GG and zein, as zein has an absorption peak at around 1655 1/cm–1645 1/cm and 1547 1/cm–1558 1/cm, which represents amide I and amide II, respectively (Zhang et al. 2019). Similar results were reported when zein/GG composite particles were used to stabilize the emulsion. The authors suggest these peaks are associated with electrostatic interaction between zein and GG (Jiang et al. 2021). Peaks at 1410 1/cm and 1726 1/cm were attributed to the C–H stretching of the methyl group and the C=O stretching of alkyl ester, respectively. Therefore, the intermolecular interaction between a film with zein and GG only involves hydrogen bonding and electrostatic interactions. After adding various ROE concentrations, the pattern of the FTIR spectrum remains very similar and there are no observable changes. This phenomenon suggests that adding ROE does not impart modifications to the structure of zein/GG film. The recent finding shows no significant changes in the FTIR spectrum of chitosan/sodium caseinate/rosemary essential oil film (di-Giuseppe et al. 2022). These indicate no chemical changes in the film structure after ROE addition.

### Physical characteristics

#### Swelling ratio and water contact angle (WCA)

The swelling ratio of active film incorporated with various ROE concentrations was investigated to determine the effect of ROE on the film’s ability to water absorption. As seen in Table 1, the film before ROE addition has a low swelling ratio (19164%). Zein, as a hydrophobic protein, can be responsible for this phenomenon because zein repels water and reduces the water uptake of the film. After the addition of ROE, a similar effect was also observed for higher ROE. Adding 15% and 20% ROE results in 18003% and 17282% swelling ratio, respectively.

**Table 1:** Swelling ratio, TSM, WCA and OP of active film incorporated with various ROE concentrations (0–20%).

ROE concentration (%)	Swelling ratio ± SD (%)	TSM ± SD (%)	WCA ± SD (°)	OP ± SD (mEq hydroperoxide/kg oil)
0	19,164 ± 1777^bc^	54.8 ± 6.0^a^	118.6 ± 2.7^a^	44.4 ± 0.3^a^
5	25,085 ± 2264^a^	50.0 ± 12.3^a^	112.6 ± 4.5^a^	42.5 ± 0.4^ab^
10	23,162 ± 511^ab^	40.7 ± 9.4^a^	115.5 ± 2.2^a^	41.3 ± 0.1^ab^
15	18,003 ± 2827^c^	59.0 ± 6.7^a^	116.7 ± 1.6^a^	42.0 ± 2.1^ab^
20	17,282 ± 1834^c^	64.4 ± 9.5^a^	112.5 ± 3.2^a^	39.4 ± 0.5^b^

*a–c represents statistically significant differences in swelling ratio, TSM, WCA and OP of active film between various ROE concentration (0–20%). The values stated are mean ± SD.*

Furthermore, the swelling ratio of the active film decreases when the ROE concentration increase. This may be due to the formation of a more compact film structure, which resulted in reduced free volume and a less permeable film matrix (Hosseini et al. 2015). This was seen from FESEM and AFM (Figure 1), where an increased aggregation between zein and ROE is observed at higher ROE concentrations. Gelatin-based packaging films incorporated with tea polyphenol and rosemary extract also reported similar results. Lower swelling properties were observed when a higher concentration of tea polyphenol or rosemary extract was added to the active films (Walid et al. 2022).

The effect of ROE on the surface hydrophobicity of active films was elucidated through the water contact angle. In general, a film surface with higher WCA represents a higher surface hydrophobicity. To quantitatively differentiate between hydrophobic and hydrophilic surfaces, WCA more than 65° is considered a hydrophobic surface, while WCA less than 65° is regarded as a hydrophilic surface (Vogler 1998). All films, with or without the addition of ROE, showed high surface hydrophobicity, as the WCA of all films is above 110°. Therefore, in this study, the addition of ROE maintained the excellent surface hydrophobicity of zein/GG film. The aggregated granules of GG may be attributed to this high surface hydrophobicity, while the presence of zein and ROE did not result in observable changes in these granules (Figure 1). Similar behavior was also seen when rosemary essential oil was added to gelatin/chitosan-based film, and no significant differences in WCA were observed while maintaining the hydrophilicity of the biocomposite film (Roy and Rhim 2022).

#### Oxygen permeability

Oxygen permeability is one of the essential characteristics in the understanding of food packaging film. In this study, the oxidation of sunflower oil was determined through peroxide value (PV), representing the active film’s ability to control oxygen transfer. Adding 20% ROE into active film significantly (*p* < 0.05) reduces the PV from 44.4 ± 0.28 to 39.44 ± 0.46 meq hydroperoxide. This indicates that active film can effectively limit oxygen transfer compared to film without ROE addition. The results suggested that oxygen molecules have to pass through a tortuous path in the active film, due to ROE addition. So, the oxygen barrier performance was enhanced. A chitosan-based film with rosemary extract added had improved oxygen barrier properties, and the oxygen transfer was reduced by 53% (Giannakas et al. 2019). It is also likely that ROE in active film has an oxygen scavenging effect. A study involving thermoplastic starch films incorporated with rice and coffee husk extract that contained antioxidant compounds also showed a 50–85% reduction in oxygen permeability, suggesting that these results are associated with the antioxidant compound (Collazo-Bigliardi et al. 2019). Thus, the increase in tortuosity and oxygen scavenging effect after ROE addition contributes to the reduction of oxygen permeability of active film.

### Antioxidant activity of active films

The antioxidant activity of active film incorporated with ROE was determined through TPC, DPPH and FRAP assay. TPC was performed to determine the total amount of polyphenol present in the active film. At the same time, DPPH and FRAP assays were done to assess the ability of active film to scavenge free radicals.

**Fig. 3:**
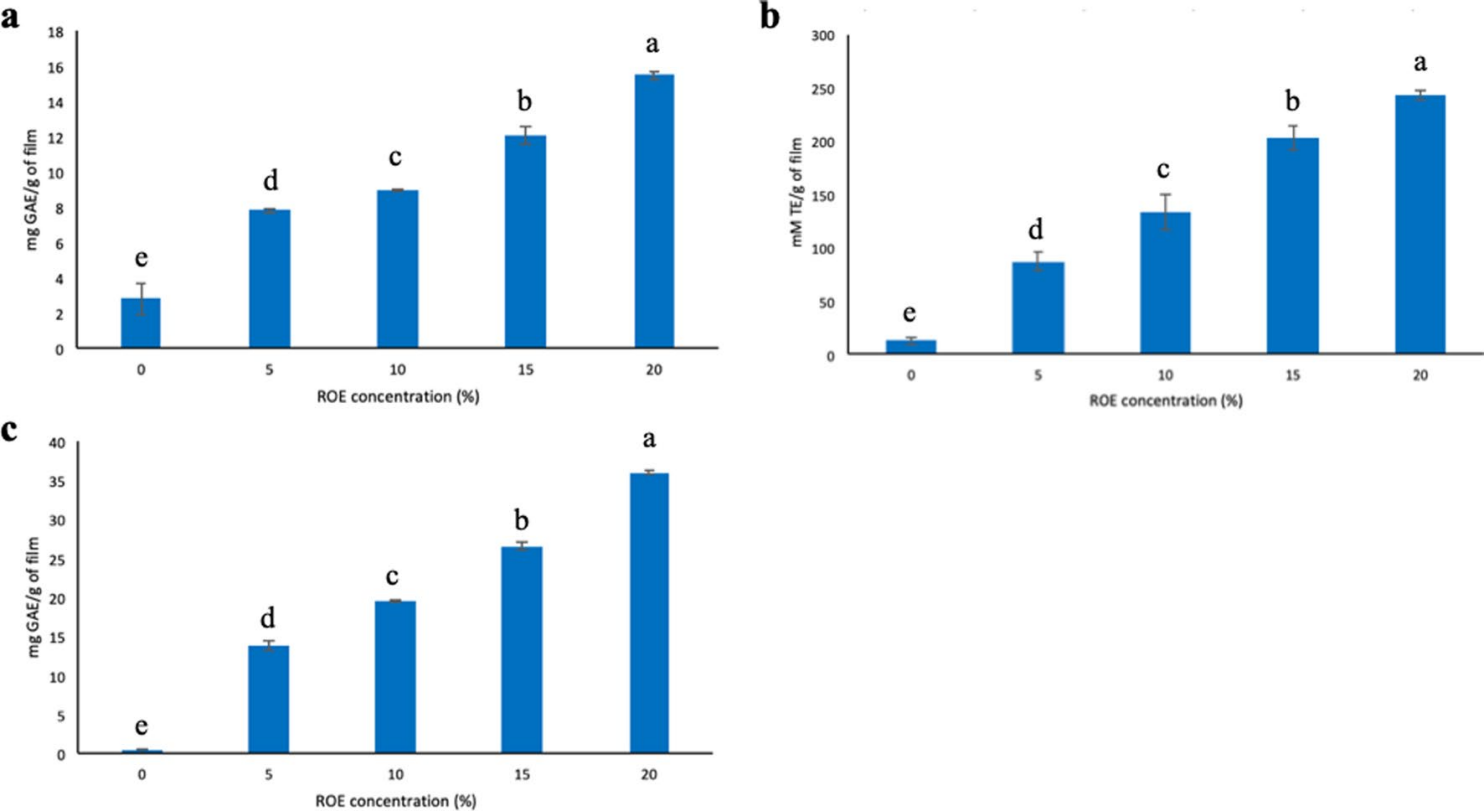
Antioxidant analysis, **a** TPC, **b** DPPH-free radical scavenging effect and **c** FRAP assay of active film incorporated with various ROE concentrations (0-20%). a–e represents statistically significant differences of TPC, DPPH-free radical scavenging effect and FRAP assay of active film between various ROE concentrations (0–20%).

In Figure 3, the TPC of the active film is significantly (*p* < 0.05) higher, with 15.47 ± 0.72 mg GAE/g of film, as compared to the film without ROE addition, with 2.81 ± 0.9 mg GAE/g of the film. This is 5.5 times higher after the addition of 20% ROE. Similarly, the addition of ROE significantly (p < 0.05) increased the DPPH-free radical scavenging ability, with 242.90 ± 1.80 compared to 13.10 ± 3.80 mM TE/g of the film without the ROE addition. On top of that, FRAP assay also showed similar results, with 30.06 ± 0.81 mg GAE/g of the film after the addition of ROE and 0.38 ± 0.20 mg GAE/g of the film without the ROE addition. Besides that, the TPC, DPPH-free radical scavenging ability and FRAP of the active film increased when the concentration of ROE increased from 5% to 20%.

The increase of TPC in the active film is primarily attributed to the polyphenols present in ROE. These polyphenol compounds exhibit strong antioxidant properties, such as carnosol, carnosic acid, and rosmarinic acid, and are extracted from rosemary plants. Therefore, this is in accordance with the excellent radical scavenging effect of the active film, whereby when the TPC increases, the DPPH-radical scavenging effect and FRAP also increase. A study that involved the addition of rosemary into chitosan film resulted in higher TPC than pure chitosan film (Abdollahi et al. 2012). Furthermore, chitosan-based film embedded with rosemary extract also exhibits high DPPH free radical scavenging activity and is closely related to the level of rosemary extract (Du et al. 2021). In conclusion, this study elucidated remarkable antioxidant effects of the active film when ROE is added into zein-loaded GG film and the potential in retarding food oxidation.

### Release behavior and diffusion coefficient (*D*) of active compound from a film into food stimulants

**Fig. 4:**
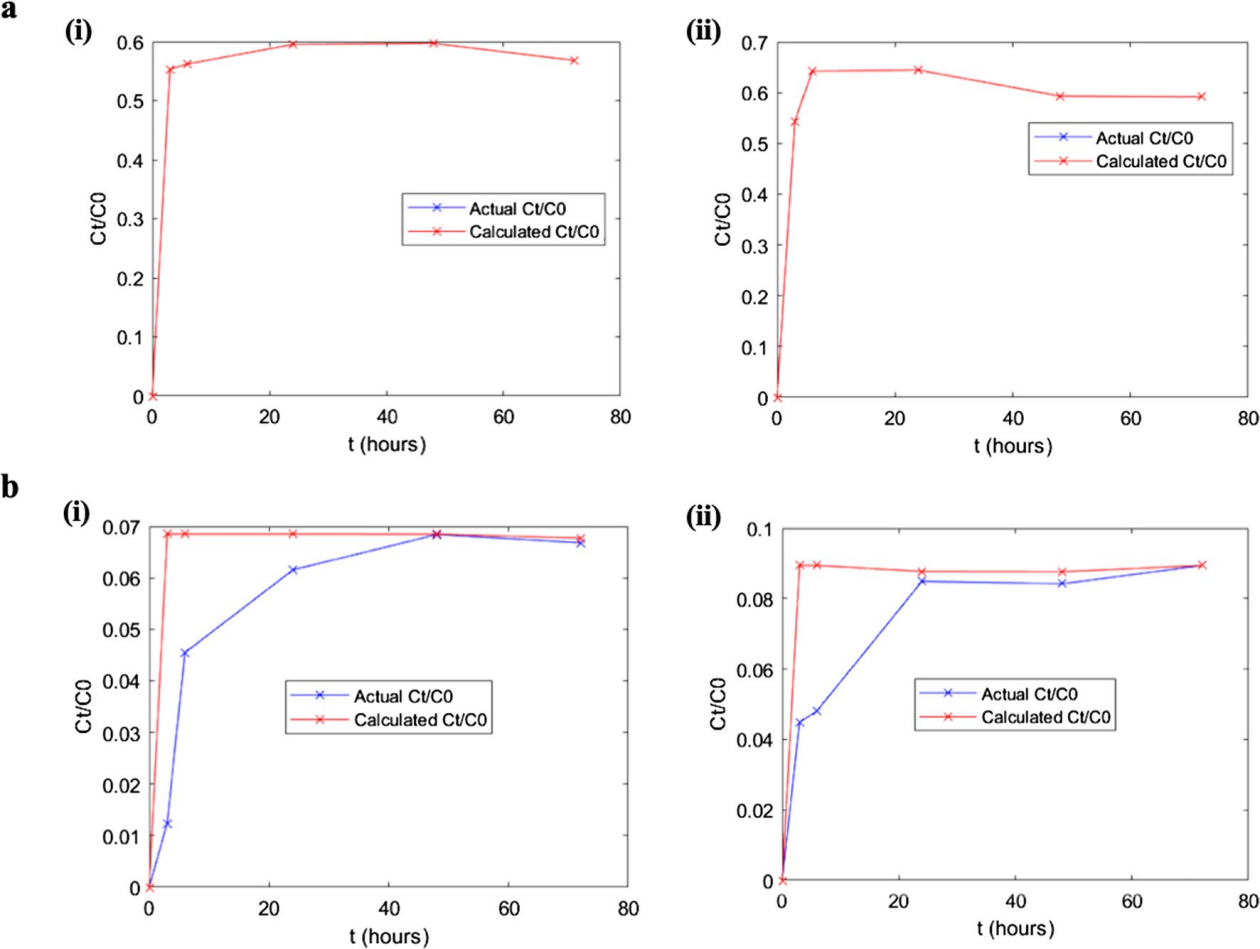
Release behavior of ROE from the active film into **a** fatty food stimulants, (i) 4 °C and (ii) 24 °C and **b** hydrophilic food stimulants, (i) 4 °C and (ii) 24 °C. The red line represents the theoretical data obtained from mathematical modeling, while the blue line represents actual experiment results.

Release tests are essential in food packaging, as this indicates the behavior of releasing active compounds from the active film into different foods. As seen in Figure 4, the release of ROE from the active film into fatty food stimulants at 4 °C and 24 °C increases from 0 to 24 h and reaches a plateau after 24 h. However, the release of ROE during equilibrium (after 24 h) is higher at 24 °C, compared to 4 °C. This phenomenon was also shown by determining *D* through mathematical modelling for active film in fatty food stimulants. From Table 2, the diffusion coefficient of ROE in 24 °C (*D* = 21.140 × 10^−14^) is higher than 4 °C (*D* = 5.115 × 10^-14^). On the other hand, the release of ROE from active film into hydrophilic food stimulants also showed a similar trend at 4 °C and 24 °C. The release of ROE reaches a maximum after 3 h and remains plateau throughout 72 h (Figure 4). Similarly, the release of ROE is also higher at 24 °C, compared to 4 °C, after equilibrium has been achieved.

**Table 2:** *D*, *α* and *R*^2^ of ROE released from an active film into fatty and hydrophilic food stimulants, under 4 and 24 °C.

Food stimulants	Temperature (°C)	*D* ± SD (10^-14^ m^2^/s)	*α* ± SD (10^−4^)	*K*_FS_ ± SD	*R*^2^
Fatty	4	5.115 ± 4.609^b,X^	147 ± 0.07^b,X^	0.681 ± 0.033^a,Y^	1.000
	24	21.140 ± 0.508^a,X^	181 ± 0.02^a,X^	0.552 ± 0.004^b,Y^	1.000
Hydrophilic	4	0.802 ± 0.210^a,X^	7.34 ± 0.24^b,Y^	13.630 ± 0.442^a,X^	0.144
	24	5.949 ± 3.340^a,Y^	9.19 ± 0.11^a,Y^	10.880 ± 0.132^b,X^	0.386

*a–b and X–Y represent statistically significant differences of D-value α and K*_FS_* of ROE in different temperatures and food stimulants, respectively. The values stated are mean ± SD.*

The ROE release is higher at 24 °C in fatty and hydrophilic food stimulants. This is because the film matrix will have greater mobility at higher temperatures resulting in greater free volume (Debot et al. 2019). Thus, food stimulants can easily penetrate the film matrix, increasing ROE release. While at lower temperatures, the film matrix will have lower mobility and reduced penetration of food stimulants into the film matrix. Similar results were reported from earlier studies, where more significant release of carotenoids from cellulose acetate films was observed for 40 °C compared to 25 °C (Assis et al. 2021). Furthermore, when sodium caseinate nanocomposite films were kept at 4 °C, 25 °C and 37 °C, the highest release of cinnamon essential oil was obtained at 37 °C (Ranjbaryan et al. 2019).

When ROE release is compared between fatty and hydrophilic food stimulants, the release is higher in fatty food stimulants than in hydrophilic food stimulants. *C*_t_*/C*_*0*_ is the ratio of ROE concentration at time *t* to initial ROE concentration in active film. In fatty food stimulants, the highest *C*_t_*/C*_*0*_ is 0.595 and 0.644 for 4 °C and 24 °C, respectively, and these values are higher than in hydrophilic food stimulants, with 0.0684 and 0.0893 for 4 °C and 24 °C, respectively. This is likely attributed to the hydrophobic nature of ROE, leading to low ROE release in the hydrophilic food stimulants. Zein is a hydrophobic protein whose hydrophobic residue can interact or bind with ROE. Like ROE, curcumin, a hydrophobic compound, can form a complex with soy protein isolate through hydrophobic interactions (Tapal and Tiku 2012). Therefore, it is highly likely that zein acts as a core to encapsulate ROE and both are released in fatty food stimulants.

Solubility and polarity between ROE in active film and food stimulants are predominant factors in controlling ROE release. Differences in food stimulants solubility and polarity will affect the ROE release from the active film (López-Córdoba et al. 2017). This is evident from Table 1, where the *K*_FS_ of ROE is significantly (*p* < 0.05) lower in fatty food stimulants at 4 °C and 24 °C. This low *K*_FS_ indicates that most ROE are released into food stimulants and may be attributed to the similar high polarity between fatty food stimulants and ROE. A study suggests that the release of a non-polar compound, limonene, increases when the ethanol concentration in food stimulants increases from 10% to 95% (Jiang et al. 2022). The highest release in 95% ethanol food stimulants is due to a reduction in bonding between the non-polar compound and hydrophilic film, as the non-polar compound has excellent solubility in a high ethanol environment (Chollakup et al. 2020).

Despite the *higher D values for ROE in fatty food stimulants* than in hydrophilic food stimulants, the comparison might not be reliable for both temperatures. This is because the *R*^2^ for fatty food stimulants is 1.000, which can be translated to a perfect model where the predicted data fit the experimental value well. This is evident in Figure 4, whereby the trendline for theoretical data and observed value overlapped. However, the R^2^ for hydrophilic food stimulants are 0.144 and 0.386, for 4 °C and 24 °C, respectively. This indicates that the predicted value did not fit well with the experimental value. This might be attributed to the low *α* value, where most of the ROE remained in the active film and the model used to calculate the experimental data is unsuitable (Cruz et al. 2008). Therefore, mathematical models that can accommodate a very low diffusion from film to food stimulants may be best suited to explain this condition.

## Conclusions

In this study, zein/GG/ROE active film was successfully fabricated, and this active film exhibited excellent antioxidant effects. The addition of ROE maintains the overall morphological structure, as FESEM and AFM showed that active film with all ROE concentrations exhibit similar film structure. Active film with ROE enhanced water resistance performance. Whereby the swelling ratio decreased significantly by 31% at high ROE concentration. This might be attributed to the hydrophobic nature of ROE. Besides, improved oxygen barrier was determined, as the OP reduced significantly with a 20% ROE addition. ROE creates a tortuous path for oxygen molecules, which impedes the movement across the active film. Active film incorporated with ROE showed excellent antioxidant activity with high TPC, excellent DPPH-free radical scavenging effect and FRAP. Higher ROE release is determined in fatty food stimulants when compared to hydrophilic food stimulants. This was determined with a higher *D* value. Furthermore, ROE release is also higher at room temperature (24 °C) than at refrigeration temperature (4 °C) in fatty and hydrophilic food stimulants. This is because the increase in temperature will result in higher energy, which enhances ROE release. Thus, this active film can potentially protect food products from oxidation, especially on high oil or fat foods, as a higher release of ROE is determined in fatty food stimulants. This active film can be used as primary packaging to pack edible oils.

## Data Availability

Data will be made available on reasonable request.

## Abbreviations

GG: Gellan gum
HAGG: High-acyl gellan gum
ROE: Rosemary oleoresin extract
D: Diffusion coefficient
TSM: Total soluble matter
WCA: Water contact angle
OP: Oxygen permeability
K_FS_: Partition coefficient between film and food stimulants
SD: Standard deviation

## Code availability

Upon request from the reviewer.
